# The Feeding of Infants

**Published:** 1894-01

**Authors:** 


THE FEEDING OF INFANTS. Hauser (*Berlin. klin. Woch.*, August 14, 1093) speaks of the successful use of the new method of feeding infants. He has used in Henoch's clinic and elsewhere, a preparation of cow's milk, introduced by Rieth, in which, after cream and milk sugar have been added to make up for the deficiency in these substances, egg albumen, heated above 130° C., is also added to supply the deficiency in albumen. The preparation has the same composition as woman's milk. When cow's milk, properly prepared and sterilized, does not agree with the child, the author substitutes this preparation. He employs it in two classes of cases, first in those in whom the cow's milk is well received, but who do not thrive, and secondly, in those who have impaired digestion Some sixty infants were treated with this preparation, and the author has now used it for one year and a half. The infants take it well, vomiting ceases and the weight increases. In bad cases it is given cold and in small quantities. The stools become regular and healthy, but are often offensive from the sulphur in the albumen. The preparation is also useful in acute diseases, rickets and other disorders attended with malnutrition. As the children grow older cow's milk may be added to the albumen milk until, at length, the former is taken pure.—*Annals Gyn. and Pæd.*

